# Fish Microbiome Modulation and Convenient Storage of Aquafeeds When Supplemented with Vitamin K1

**DOI:** 10.3390/ani12233248

**Published:** 2022-11-23

**Authors:** Marcos Acosta, Eduardo Quiroz, Dariel Tovar-Ramírez, Vânia Palma Roberto, Jorge Dias, Paulo J. Gavaia, Ignacio Fernández

**Affiliations:** 1 Centro de Investigaciones Biológicas del Noroeste, Av. Instituto Politécnico Nacional 195, Col. Playa Palo de Santa Rita Sur, La Paz 23096, BCS, Mexico; 2 CONACYT-CIBNOR, Av. Instituto Politécnico Nacional 195, Col. Playa Palo de Santa Rita Sur, Baja California Sur, La Paz 23096, BCS, Mexico; 3 ABC Collaborative Laboratory, Association for Integrated Aging and Rejuvenation Solutions (ABC CoLAB), 8100-735 Loulé, Portugal; 4 Algarve Biomedical Center Research Institute (ABC-RI), Campus Gambelas, Bld.2, 8005-139 Faro, Portugal; 5 SPAROS Ltd., Área Empresarial de Marim, Lote C, 8700-221 Olhão, Portugal; 6 Centro de Ciências do Mar (CCMAR), Campus de Gambelas, University of Algarve, 8005-139 Faro, Portugal; 7 Associação Oceano Verde–GreenCoLab, Campus de Gambelas, University of Algarve, 8005-139 Faro, Portugal; 8 Centro Oceanográfico de Vigo, Instituto Español de Oceanografía (IEO), CSIC, 36390 Vigo, Spain

**Keywords:** phylloquinone, fat-soluble vitamins, temperature, *Solea senegalensis*, pathogens, flatfish

## Abstract

**Simple Summary:**

Vitamin K (VK), and particularly phylloquinone (VK1), is an essential micronutrient whose stability in aquafeeds has not been extensively evaluated. Losing stability can lead to nutritional deficiency, which is known to hamper fish development and physiology. Gut microbiota also plays a key role in host health through the interaction with several biological processes. The present study evaluated the best storing conditions of aquafeeds when supplemented in VK1 and intestinal microbiota modulation in Senegalese sole (*Solea senegalensis*) juveniles. Aquafeeds with a high level of VK1 supplementation required storage at −20 °C for short-term (up to 7 days) and at −80 °C for long-term (up to three months) to ensure optimal preservation. Furthermore, gut bacterial communities of Senegalese sole specimens fed with a commercial feed supplemented with VK1 showed a better-balanced population of microorganisms in the intestine, which might improve Senegalese sole health during the ongrowing phase. These results provide the practical guidelines for the proper storing of aquafeeds in the industry when supplemented with VK1 and highlight the potential benefits of dietary VK1 supplementation for a balanced intestinal microbiota and overall fish health.

**Abstract:**

Vitamin K (VK) is a fat-soluble vitamin necessary for fish metabolism and health. VK stability as dietary component during aquafeed storage and its potential effect on intestinal microbiome in fish have not yet been completely elucidated. The convenient storage conditions of aquafeeds when supplemented with phylloquinone (VK1), as well as its potential effects on the gut microbiota of Senegalese sole (*Solea senegalensis*) juveniles, have been explored. Experimental feeds were formulated to contain 0, 250 and 1250 mg kg^−1^ of VK1 and were stored at different temperatures (4, −20 or −80 °C). VK stability was superior at −20 °C for short-term (7 days) storage, while storing at −80 °C was best suited for long-term storage (up to 3 months). A comparison of bacterial communities from Senegalese sole fed diets containing 0 or 1250 mg kg^−1^ of VK1 showed that VK1 supplementation decreased the abundance of the *Vibrio*, *Pseudoalteromonas*, and *Rhodobacterace* families. All these microorganisms were previously associated with poor health status in aquatic organisms. These results contribute not only to a greater understanding of the physiological effects of vitamin K, particularly through fish intestinal microbiome, but also establish practical guidelines in the industry for proper aquafeed storage when supplemented with VK1.

## 1. Introduction

Aquaculture is a fast-growing industry worldwide and represents an important source of food for the human population [1]. Many species of marine fish are farmed on a commercial scale. Senegalese sole (*Solea senegalensis*, Kaup 1858) is a common flatfish species distributed in the Central Atlantic (from France to Angola coast), with an oval and asymmetric body, living in sandy or muddy bottoms up to a depth of 100 m. There is great farming potential on a commercial scale due to its high consumer acceptance and economic value [2]. However, sole farming is still facing different production limitations. Nutrition and disease outbreaks during the ongrowing phase are among the major constraints [2]. Indeed, satisfying the nutritional and physiological demands of this species is one of the main challenges to reach a better welfare status within the industry [3].

Fulfilling nutritional requirements for micronutrients is an essential part of fish nutrition and health outcomes, but the functions and doses of particular micronutrients have not yet been fine-tuned for many important commercial species [4]. This is the case for vitamin K (VK), which, as many other fat-soluble vitamins, is necessary for fish since they are not capable of de novo synthesizing it (reviewed in [5]). The VK can be found in three distinct forms: Phylloquinone (VK1), menaquinone (VK2) and/or menadione (VK3; [6]). VK1 and VK2 are natural forms produced by green plants and microorganisms, respectively [5]. In contrast, VK3 is artificially synthesized, and is the most frequently used form of VK in animal feeds (particularly for fish species), either as menadione sodium bisulfite (MSB) or as menadione nicotinamide bisulfite (MNB; [5,7]). While both VK3 and VK1 are unstable compounds (as many other vitamins), VK1 has shown better attributes in terms of biological functionality, as well as less toxicological effects than VK3 commercial forms [8,9,10]. The preferential use of one VK form in aquafeeds is mainly due to production system and costs. VK1 production requires chemical synthesis from VK3 and/or complex and less optimized biotechnological processes [11].

VK is historically known for being essential for blood coagulation control, although its role in skeletal development, glucose metabolism, reproduction, and cognitive capacities has been recently evidenced (reviewed in [5]). Its deficiency has been shown to dramatically affect fish development, growth and redox balance through different molecular pathways [12,13,14,15,16]. Regardless of the source and their functions, K vitamers can be susceptible to significant losses due to chemical and physical conditions during technological processing or storage [17]. Therefore, the reduced stability of VK forms can result in inadequate nutrient content, leading to the reduced health and quality of produced animals. Thus, to successfully implement the use of VK1 as a dietary additive in aquafeeds, it is necessary to identify the appropriate storage conditions of aquafeeds. In particular, the determination of optimal time and temperature conditions for storing feeds supplemented with VK1 is critical to warrant their beneficial effect in fish farming industry.

During the last decade, VK1 has been reported as an interesting K vitamer source in feed formulations to improve fish skeletal development, reducing the incidence of skeletal deformities [18] and improving fish sperm quality [19]. In eukaryotic cells, VK can act as electron and proton transporters, as well as in the cell antioxidant response upon stress conditions [20,21]. Some prokaryotes can reduce ubiquinone or K substrates [22], and recent works showed that some vitamin K forms are considered “growth promoter factors” for bacteria that have lost the ability to synthesize quinones but preserve the capacity to metabolize them from external sources [23]. In this sense, intestinal microbiome is now recognized as an extremely important biological system interacting with the host [24,25] thanks to the high throughput 16s rRNA gene sequencing technologies, allowing for the molecular identification of previously undetectable bacteria due to culture limitations [26]. Like in other vertebrates, fish microbiome is linked to host health through biological processes such as nutrient processing, detoxification, modulation of intestinal motility, immune and disease resistance, as well as development and maturation of the mucosa [24,27,28]. Moreover, the gastrointestinal microbial composition has complex and dynamic interactions with the environment and can also be influenced by several factors, including diet and feeding habits [29].

The potential effect of dietary components on the composition of bacterial communities is a line of research with growing relevance [30]. Dietary components shape the gut microbiome by modulating the abundance of specific species and their individual or collective functions [31]. Until now, several studies have explored the effect of diet on the fish microbiome [32,33,34]. However, very few have identified if (and how) dietary VK content affect vertebrate’s microbiome [35], and none have been exclusively focused on fish species.

The present study aimed at filling the research gaps on convenient aquafeed storing conditions when aquafeed is supplemented with VK1, as well as to evaluate the potential effect of this supplementation on the intestinal microbiota of Senegalese sole.

## 2. Materials and Methods

### 2.1. Ethical Statement

All the people involved in the experiments have a FELASA class C permit for animal experimentation. Fish facilities (permit 0421/2013), personnel and experiments (permit 0421/2020) were accredited and approved by the Portuguese National Authority for Animal Health (DGAV). All experiments complied with the ARRIVE guidelines [36] and were performed according to 2010/63/EU of the European Parliament and Council, guideline 86/609/EU of the European Union Council and Portuguese legislation (Decreto-Lei 113/2013) for animal experimentation and welfare.

### 2.2. Development of Experimental Diets and Quantification of Dietary VK Levels under Different Storage Conditions

Three experimental isolipidic and isonitrogenous semi-dry fish feeds were developed by Sparos Lda (http://www.sparos.pt/index.php/en/; Olhão, Portugal) with equal proximate composition of crude protein (62.9%), crude fat (14.8%), crude fiber (1.1%), crude ash (10.2%) and total phosphorus content (1.5%). Experimental feeds were specifically formulated to contain different levels of VK1 (Sigma-Aldrich, Nº Cat: V3501, CAS nº 84-80-0): not supplemented with VK1, Control diet (Control); diet supplemented with 250 mg kg^−1^ of VK1 (VK250); and diet supplemented with 1250 mg kg^−1^ of VK1 (VK1250). Diets were protected from light with opaque bags and in vacuum before opening. Equal amounts of diets (5 kg) were stored at 4, −20 or −80 °C for 7 days, 4 weeks or 3 months, respectively. The experimental design and sampling times are depicted in the upper panel of Figure S1. When stored at 4 °C, VK content in feeds was evaluated at 1, 3 and 7 days after the feed bag was opened. When stored at −20 °C, the VK content was assessed at 1, 2, 3 and 4 weeks; while at −80 °C the VK levels were evaluated at 1, 2 or 3 months. Dietary VK content was evaluated in two different feed subsamples through HPLC with fluorescence detection analysis (Silliker-Mérieux NutriSciences, France; https://www.merieuxnutrisciences.com/ (accessed on 16 November 2022); Vila Nova de Gaia, Portugal).

### 2.3. Fish Rearing and Maintenance

Senegalese sole eggs obtained from natural spawning of the broodstock at the EPPO/IPMA Aquaculture Research Station (Olhão, Portugal) were transferred to Centro de Ciências do Mar (CCMAR)—Universidade do Algarve (Faro, Portugal) for thermal, light and chemical acclimatization. After incubation in a semi-closed water recirculation system equipped with mechanical and biological filters, protein skimmer and UV sterilizer, hatched larvae were randomly distributed in four 100 L white cylindrical-conical tanks with a density of 95 larvae L^−1^. At 19 days after hatching (DAH), post-larvae were transferred to 3-L flat-bottomed plastic trays (120 specimens per tray). The environmental parameters were: 19.9 ± 1.2 °C, 36.9 ± 1.2 g of salinity L^−1^, 97.6 ± 4.9% of dissolved oxygen saturation, 12 h of light: 12 h of dark photoperiod and 900 lux of light intensity at the water surface. 

Larvae were progressively fed three times a day with live prey: Rotifers (*Brachionus rotundiformis*) from 2 to 10 DAH, *Artemia* nauplii (AF strain; INVE, Dendermonde, Belgium) from 6 to 10 DAH, and *Artemia* metanauplii (EG strain; INVE) enriched with Red Pepper^TM^ (Bernaqua, Belgium) from 9 to 17 DAH. The same *Artemia* metanauplii, but immediately frozen after enrichment, were provided to larvae from 18 to 30 DAH. Larvae were subjected to progressive co-feeding until weaning was completed. Specimens were fed with an inert diet (https://www.sparos.pt/products/#winflat (accessed on 16 November 2022)), specifically designed for the Senegalese sole by Sparos Lda, until five months.

### 2.4. Experimental Design

A total of 46 fish juveniles of 48.3 ± 16.0 g of the mean wet body weight were randomly distributed in two flat bottom 200 L tanks (23 specimens each) connected to a single recirculating aquaculture system. Environmental parameters of rearing conditions were as described above. Based on the results of dietary VK1 levels under different storage conditions and time (see Results Section 3.1), fish were fed with two experimental diets: A control diet without VK1 supplementation (CT) or a diet supplemented with 1250 mg kg^−1^ of VK1 (VK1250) properly stored at −80 °C (when to be used for more than 1 week) and −20 °C (when to be used in less than 7 days) along the experiment. Diet supplemented with 1250 mg kg^−1^ of VK1 was selected based on previous results on the same species [19]. Feed production was carried out by the team at Sparos Lda, with expertise and facilities for feed formulation and production under current standard methods used at the commercial scale. The nutritional trial lasted 30 days and the fish were fed once per day with 3% daily feed intake.

The ammonium and nitrite water concentrations were daily monitored to keep them below toxic values. The experimental design and sampling times are depicted in the bottom panel of Figure S1. Four fish were sampled at the start of the experiment (CT0), while the other four fish from each experimental group (CT30 and VK30) were sampled at the end of the experiment (30 days). 24 h fasting specimens were previously euthanized with an overdose (0.3 g L^−1^) of tricaine methanesulfonate (MS-222, Sigma-Aldrich, Madrid, Spain). Fish were weighted, its whole intestine dissected, and its microbiota collected and stored in 100% ethanol at −80 °C until processed for DNA library preparation and sequencing.

### 2.5. Isolation, Library Preparation and Sequencing

Total DNA was isolated from each sample using the PowerSoil^®^ DNA Isolation Kit (Catalogue nº 12888-50; Mo Bio Laboratories Inc., Carlsbad, CA, USA) according to the manufacturer’s protocol. Libraries preparation was performed using the amplification of the V4 regions of the 16S rRNA gene with the primers set 515F (5′-GTGCCAGCMGCCGCGGTAA-3′) and 806R (5′-GGACTACHVGGG TWTCTAAT-3′) following the method described in [37]. The size, purity, and concentration of the libraries were assessed using the D1000 High Sensitivity ScreenTape Assay (Agilent, Santa Clara, CA, USA). Normalized libraries were pooled and amplicons (end pairs: 300 × 300 bp) were sequenced in the Illumina MiSeq platform by MR DNA (www.mrdnalab.com, Shallowater, TX, USA). All sequencing data were submitted to the NCBI SRA database (accession number PRJNA875592).

### 2.6. Bioinformatic Analysis

Obtained 16S rRNA gene sequences were analyzed using MOTHUR software v1.35.1 from the University of Michigan; Ann Arbor, MI, USA (Schloss et al. 2009), according to that previously described by [38]. Briefly, paired-end reads were merged, quality-filtered, and aligned using the SILVA v138 (Microbial Genomics and Bioinformatics Group at the Max Planck Institute for Marine Microbiology, Germany) database [39]. Sequences were then randomly subsampled (to normalize the number of sequences) and assigned to operational taxonomic units (OTUs) based on a 97% sequence similarity. Alpha diversity was calculated using the Shannon diversity index (H′) and the Chao richness estimator. Reference alignment was performed with RDP (v.9) (Michigan State University Board of Trustees, East Lansing, MI, USA) [40].

### 2.7. Statistical Analysis

Data is presented as mean ± standard deviation (SD) and was checked for normality (Shapiro-Wilk test) and equal variances (F-test). Dietary VK1 levels were compared by means of one-way ANOVA, and when significant differences were detected with a Tukey post-hoc analysis between (i) experimental diets at each sampling time and storing condition and (ii) along time for each experimental diet and storage condition. Abundance at the family and genus level was compared using the one-tailed *t*-test or Welch’s *t*-test, depending on whether there were equal variances or not. Two separate analyzes were performed to determine differences between groups fed with different dietary levels of VK1 (CT30 and VK30) and to establish if there was a difference between sampling times (CT0 and CT30). Regarding the analysis of the composition and structure of the bacterial community, a principal component analysis (PCA) and a principal coordinate analysis (PCoA) (Bray-Curtis dissimilarity index) were conducted using GraphPad Prism 9.0 (GraphPad Software, Inc., San Diego, CA, USA). In addition, an Analysis of Molecular Variance (AMOVA) test, implemented in the MOTHUR software package v1.35.1 from The University of Michigan; Ann Arbor, MI, USA) [41], was applied to establish if the grouping within the ranges was statistically significant. Statistical significance was set to 0.05.

## 3. Results

### 3.1. Dietary VK1 Levels and Their Maintenance under Different Storage Conditions

**Figure 1:**
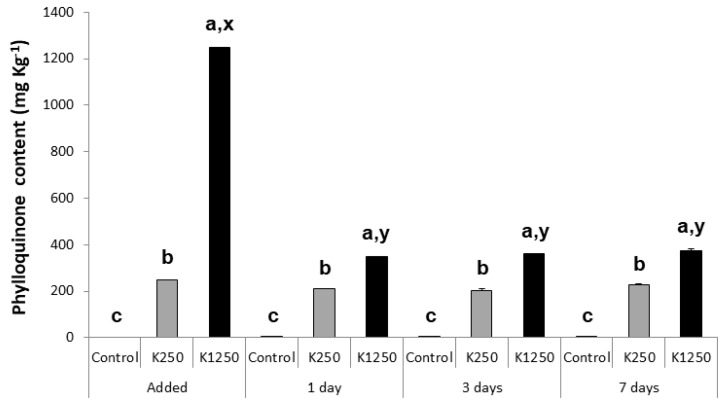
Profiles of dietary VK1 content (mean ± standard deviation) in experimental feeds when stored at 4 °C. Control, diet not supplemented with VK1; VK250, diet supplemented with 250 mg kg^−1^ of VK1; and VK1250, diet supplemented with 1250 mg kg^−1^ of VK1. Note that a, b and c letters at the top of each bar denote significant differences between the experimental diets at each sampling time, while x and y letters denote significant differences between sampling times within each experimental diet (ANOVA, *p* < 0.05, n = 2).

**Figure 2:**
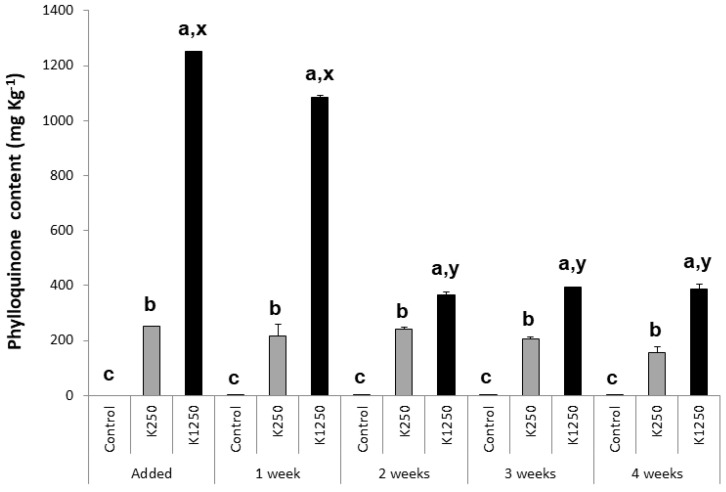
Profiles of dietary VK1 content (mean ± standard deviation) in experimental feeds when stored at −20 °C. Control, diet not supplemented with VK1; VK250, diet supplemented with 250 mg kg^−1^ of VK1; and VK1250, diet supplemented with 1250 mg kg^−1^ of VK1. Note that a, b and c letters at the top of each bar denote significant differences between the experimental diets at each sampling time, while x and y letters denote significant differences between sampling times within each experimental diet (ANOVA, *p* < 0.05, n = 2).

**Figure 3:**
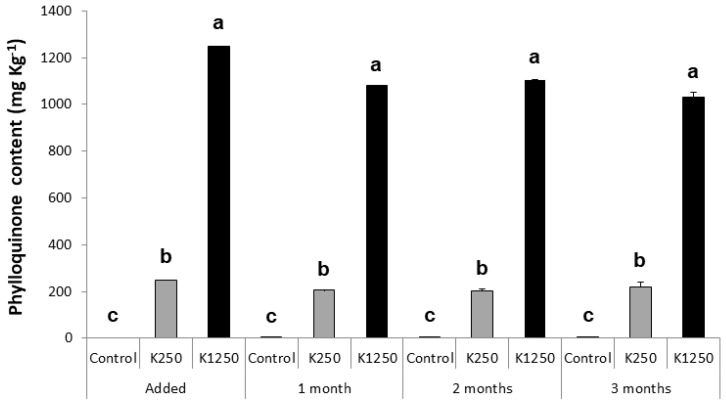
Profiles of dietary VK1 content (mean ± standard deviation) in experimental feeds when stored at −80 °C. Control, diet not supplemented with VK1; VK250, diet supplemented with 250 mg kg^−1^ of VK1; and VK1250, diet supplemented with 1250 mg kg^−1^ of VK1. Note that a, b and c letters at the top of each bar denote significant differences between the experimental diets at each sampling time (ANOVA, *p* < 0.05, n = 2). No significant differences were detected between sampling times within each experimental diet (ANOVA, *p* > 0.05, n = 2).

The dietary VK1 content of the Control, VK250 and VK1250 diets was explored when stored at different temperatures and different periods of time (Figure 1, Figure 2 and Figure 3). Independently of the storing temperature and time, VK1 content in the Control diet ranged from 0 to 0.4 ± 0.01 mg VK1 kg^−1^. Dietary VK1 levels were not significantly altered in the Control and VK250 diets when stored at 4 °C during 1, 3 or 7 days (Figure 1). However, dietary VK1 level in VK1250 sharply decreased when maintained 1 day at 4 °C, with a 72% of loss of the nominally supplemented VK1 level (from 1250 to 348.5 ± 2.12 mg VK1 kg^−1^). Such a level of VK1 was not further altered with longer (3 or 7 days) storage at 4 °C. Similarly, dietary VK1 levels in Control and VK250 diets were not significantly modified when stored at −20 °C for up to 4 weeks (Figure 2). At this temperature, high dietary VK1 levels (1085.1 ± 5.72 mg VK1 kg^−1^) were maintained for at least 1 week in VK1250 diet. Nevertheless, dietary VK1 levels in the VK1250 diet dropped to 70.7% when kept for two weeks at this temperature. The level of VK1 remained constant after the second week of storage, at least, up to 4 weeks. At −80 °C (Figure 3), no significant differences were found regarding the content of VK1 in the three experimental diets independently of the storage period tested (up to 3 months). In both VK250 and VK1250 diets, the maximal loss of supplemented VK1 were lower than 20% (19.6 and 17.5%, respectively).

### 3.2. Mothur Analysis for Sequencing Data

A total of 1,394,036 raw reads were obtained for both the forward and reverse directions. After quality trimming and filtering, the library size of each sample was normalized to the smallest number of sequences obtained from the *S. senegalensis* digestive tract samples. A total of 278,367 sequences with a mean of 18,407 ± 9176 processed sequences per sample (Table 1) and a total of 35,705 operational taxonomic units (OTUs) with 97% genetic similarity were used for subsequent analysis.

**Figure 4:**
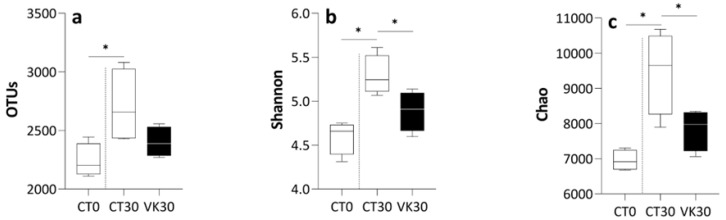
Alpha diversity values for experimental groups and sampling times. Observed OTUs (**a**), Shannon diversity (**b**), Chao richness index (**c**) in the digestive tract of *Solea senegalensis*. CT0, control group at the beginning of the trial; CT30, control group fed with the diet not supplemented with VK1 at day 30 of the trial; and VK30, group fed with the diet supplemented with 1250 mg kg^−1^ of VK1 at day 30 of the trial. Note that asterisks on the boxplots indicate significant differences between experimental groups, two separate comparisons were performed: CT30–CT0 and CT30–VK30 (*t*-test; *p* < 0.05, n = 4).

Comparison of alpha diversity estimates, regarding the observed OTUs, Shannon index and Chao richness between CT30–CT0 and CT30–VK30 showed some differences (Figure 4). In particular, a higher number of observed OTUs in CT30, when compared to the CT0 sampling time, were found. Nevertheless, no significant differences in the number of observed OTUs were observed when comparing the effect of increased dietary VK content after 30 days (VK30 vs. CT30; Figure 4a). However, the Shannon index and Chao richness were higher in the CT30 samples when compared to both CT0 and VK30 (Figure 4b,c).

### 3.3. Taxonomic Composition of Solea Senegalensis Intestinal Microbiota

**Figure 5:**
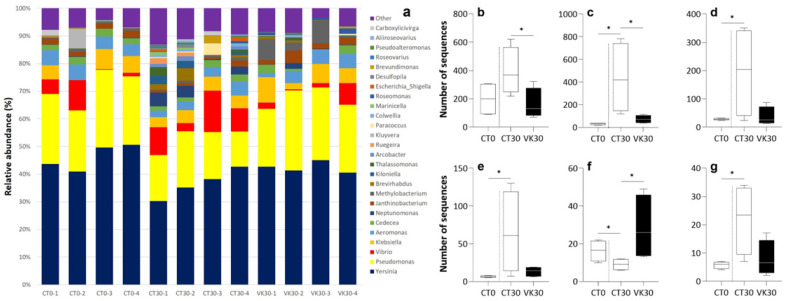
Relative and differential abundance of bacterial communities at the family level. Relative abundance (%) of the overall most prevalent families in the digestive tract of individual fish (**a**). Differential abundance (number of sequences) at the family level in the digestive tract of *Solea senegalensis*: Vibrionaceae (**b**), Rhodobacteraceae (**c**), Oceanospirillacae (**d**), Campylobacteraceae (**e**), Comamonadaceae (**f**), and Pseudoalteromonadaceae (**g**). CT0, control group at the beginning of the trial; CT30, control group fed with the diet not supplemented with VK1 at day 30 of the trial; and VK30, group fed with the diet supplemented with 1250 mg kg^−1^ of VK1 at day 30 of the trial. Note that asterisks on boxplots indicate significant differences between experimental groups, two separate comparisons were performed: CT30–CT0 and CT30–VK30 (*t*-test; *p* < 0.05, n = 4).

**Figure 6:**
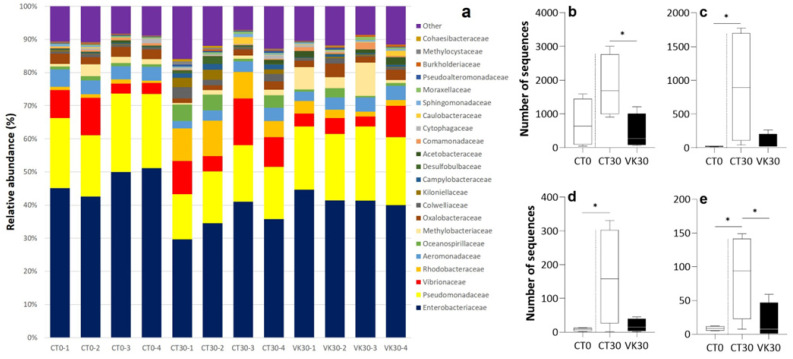
Relative and differential abundance of bacterial communities at the genus level. Relative abundance (%) of the overall most prevalent genera in the digestive tract of individual fish (**a**). Differential abundance of genera in the digestive tract of *Solea senegalensis*: *Vibrio* (**b**), *Neptunomonas* (**c**), *Colwellia* (**d**), and *Pseudoalteromonas* (**e**). CT0, control group at the beginning of the trial; CT30, control group fed with the diet not supplemented with VK1 at day 30 of the trial; and VK30, group fed with the diet supplemented with 1250 mg kg^−1^ of VK1 at day 30 of the trial. Note that asterisks on boxplots indicate significant differences between experimental groups, two separate comparisons were performed: CT30–CT0 and CT30–VK30 (*t*-test; *p* < 0.05, n = 4).

A microbiota taxonomic classification analysis was carried out at the level of the phyla, class, family and genus of those groups where their abundance represented at least 0.02% of the total composition. Proteobacteria was the most abundant phylum (ranging from 93% to 97%), Gammaproteobacteria (70–88%) the dominant class, followed by Alphaproteobacteria (3–17%) and Betaproteobacteria (1–5%). Other phyla such as Bacteroidetes, Fusobacteria and Firmicutes were detected with lower values in the microbiota of all samples (Figure S2). When the microbiota composition was analyzed at the family level, Enterobacteriaceae (35–51%) and Pseudomonadaceae (15–23%), both belonging to the Gammaproteobacteria class, were the most representative families (Figure 5a). Overall, a total of 246 genera were identified. Of these, 26 were above the detection threshold of relative abundance percentages. Specifically, *Yersinia* (30–50%) and *Pseudomonas* (12–28%) were dominant, followed by *Vibrio* and *Klebsiella* (Figure 6a).

Differences in the main bacterial families and genera of *S. senegalensis* gut were represented (Figure 5 and Figure 6). A statistical analysis was performed to detect differences between VK dietary levels, showing at family-level higher abundance of Vibrionaceae (*p* = 0.031) (Figure 5b), and Comamonadaceae (*p* = 0.034) (Figure 5f) in VK30 specimens when compared to those of CT30. In contrast, the abundance of Oceanospirillacae (*p* = 0.041) (Figure 5d), Campylobacteraceae (*p* = 0.038) (Figure 5e), and Pesudoaltermonadaceae (*p* = 0.038) (Figure 5g) were found to be significantly higher in CT30 samples than in CT0 samples. In addition, Rhodobacteraceae was the only family that denoted a greater presence in CT30 samples compared to both CT0 and VK30 (*p* < 0.05) (Figure 5c). Furthermore, significant variations in bacterial community composition were detected at the genus level. *Vibrio* (*p* = 0.0219) (Figure 6b) and *Pseudoalteromonas* (*p* = 0.046) (Figure 6e) decreased in fish fed with a VK1250 supplemented diet at day 30 (VK30), whereas the CT0 and CT30 comparison showed a higher abundance of *Neptunomonas* (*p* = 0.042), *Colwellia* (*p* = 0.037) and *Pseudoalteromonas* (*p* = 0.023) at the end of the trial (CT30) (Figure 6c–e).

### 3.4. Bacterial Community Structure

**Figure 7:**
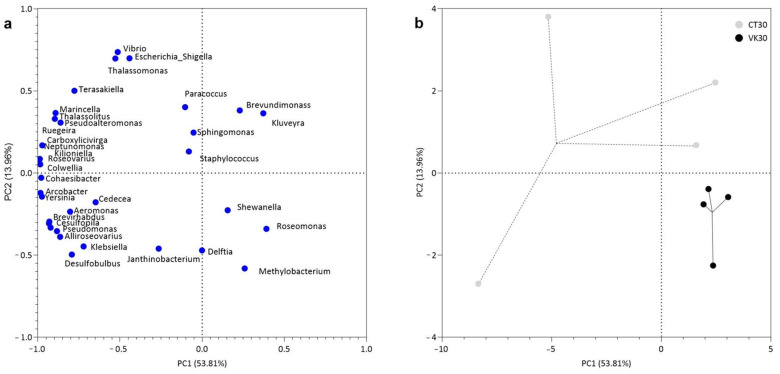
Principal component analysis (PCA) of the relative abundance of bacterial communities at the genus level. Loading graph, represents the distribution of the different genera present in the experimental groups (CT30 and VK30) (**a**). Scores graph, represents the dispersion of the samples of each experimental group (CT30 and VK30) (**b**). CT30, control group fed with the diet not supplemented with VK1 at day 30 of the trial; and VK30, group fed with the diet supplemented with 1250 mg kg^−1^ of VK1 at day 30 of the trial. In both graphs, the first and second axes represent 53.81% and 13.96% of the variation, respectively.

A principal component analysis (PCA) at the genus level was conducted to reduce the dimensionality of the dataset and to determine which genera might explain the greater variation between CT30 and VK30 treatments. With this exploratory approach, a clear separation was observed in both groups, where the first two components explained 67.7% of the total variation. Component 1 (PC1) described the highest percentage of difference, with the genera located at the first and third quadrants being more closely related with CT30 samples, while *Delftia*, *Shewanella*, *Rosemonas* and *Methylobacterium* (at the fourth quadrant) indicating an association with the VK30 samples (Figure 7a). Furthermore, a compact clustering in VK30 samples (fourth quadrant) was shown, as compared to the CT30 samples that had a wider variety in their distribution (Figure 7b).

## 4. Discussion

In the process of deciding whether a raw material or specific nutrient/additive should be included and/or supplemented in fish diets, different evaluations have been proposed [42,43]. Nevertheless, only very few studies have explored the nutrient stability and optimal storage conditions when included in commercial aquafeeds [44,45]. This assessment is critical to implement the effective use of nutrients and feed additives at the farm level. Regarding VK, several articles revealed the biological function and the associated mechanisms in fish species [12,13,14,15,16,18,19,46]. However, little is known on how storing conditions might affect their maintenance in the short and long term [47,48,49], which is basic in providing practical guidelines to the industries.

### 4.1. Thermal Storing Conditions Affect Supplemented VK1 Levels in Aquafeeds

Vitamins are relatively unstable and are susceptible to significant losses under normal storing conditions and under particular feed formulations [47,48]. Biological activity loss can be due to the formation of stereoisomers or by oxidation [49] induced by factors such as humidity (moisture), light, heat, pH, and/or exposure to oxidizing agents [48]. Specifically, K vitamers are highly sensitive to light and alkaline medium but are stable in slightly acidic media and under oxidizing conditions [17]. Temperature is one of the factors that affects its stability during feed processing and storage [50], although microencapsulation has been shown to protect it [51]. Some studies determined the effects of thermal processes and storage on VK content in feed or vitamin premixes under different conditions [51,52,53,54,55,56]. However, this is the first study that has explored VK1 content in diets when stored at different temperatures. 

Stability comparisons have shown better results at lower temperatures. Wang et al. [56] obtained a higher retention of VK3 in vitamin premixes at lower temperatures and relative humidity (RH) when comparing 25 °C/60% RH and 40 °C/75% RH for 6 months. Similarly, the stability of VK2 (menaquinone-7) has been altered when comparing storage at 25 °C/60% RH and 40 °C/75% RH and when including different mineral formulations [53]. A better stability of VK2 at 12 months when stored at lower temperatures-RH with a formulation including calcium carbonate were also reported. These studies may suggest that the preservation of VK stability may also be dependent on the formulation in which it is found, as it has also been shown that choline has negative effects on VK3 retention during vitamin premix storage [56].

Dietary profiles of VK1 in aquafeeds stored at different temperatures were studied here. While storage at 4 and −20 °C resulted in stable values of VK when supplemented with 250 mg kg^−1^ of VK1, when diets included higher supplementation levels (1250 mg kg^−1^ of VK1), immediate losses were observed after 1 day and two weeks when stored at 4 and –20 °C, respectively. In contrast, no significant differences in the phylloquinone content were found after 5 months when human milk was stored in the dark at −20 °C [57]. These discrepancies might be due to the huge difference in the total levels of VK1 present in both matrices: an approximate value of 2.5 ng mL^-1^ in human milk and 1250 mg kg^−1^ of VK1 in aquafeeds. Furthermore, while VK1 in milk might be associated with protein or lipoproteins to prevent its oxidation [58], VK1 included in aquafeeds is freely available. Indeed, lower content of VK along time seemed to be in line with its action as antioxidant [15,21], perhaps preventing lipid peroxidation. However, samples kept at −80 °C showed no loss of VK dietary levels, even when stored for up to three months. These results are in line with those reported in [59], reporting stable plasma phylloquinone levels for 12 years when frozen at −80 °C.

According to the results obtained in the present study, once the bag is opened, the feed preservation at −20 °C is the best alternative to maintain the dietary content of VK1 for a short time, while it is preferable to freeze at −80 °C for long-term storage (up to three months). Therefore, industries might require to install low-temperature storing devices in order to use diets highly supplemented in VK1 and/or more frequent acquisition of feeds, while better suited feed formulations are designed to avoid VK1 losses.

### 4.2. Supplementation of VK1 in Aquafeeds Improves Fish Microbiome Balance

In recent years, gut microbiome has been shown to have a determining role in animal health and welfare, particularly in the development and maturation of the mucosa, immunity and disease resistance [27,60,61]. On the one hand, diet and feed additives are known to exert selective pressure on the gastrointestinal microbial composition in vertebrates, including fish [29,61]. On the other hand, an imbalanced microbiome may contribute to disease development and progression, and thus it can be crucial to aquaculture success [24]. Therefore, a better understanding of how fish microbiota is modulated might unveil key mediators between microbial interactions and animal health.

Previous studies about Senegalese sole microbiota have mainly focused on determining the effects of supplementation with probiotics, prebiotics, parabiotics, immunostimulants, algal extracts or physicochemical parameters in water [62,63,64,65,66,67,68,69,70,71,72]. Present results showed that Proteobacteria and Gammaproteobacteria were the most abundant phylum and class, respectively. This is in agreement with Tapia-Paniagua et al. [63] who found this phylum to be dominant in the anterior and posterior section of the digestive tract of Senegalese sole juveniles, although Domínguez-Maqueda et al. [62] only report its dominance in the anterior section. Other phyla such as Bacteroidetes, Firmicutes, and Fusobacteria were detected with lower relative abundances in the present study, also reported to represent up to 90% of the intestinal microbiota of different fishes [26], suggesting that these taxa are involved in important functions of the host, such as digestion, absorption of nutrients and/or the immune response. Bacteroidetes and Firmicutes are known to participate in the degradation of organic matter with high molecular weight due to the action of hydrolytic enzymes, such as proteases, lipases, or amylases. Meanwhile, Fusobacteria is a group of opportunistic microorganisms that are often associated with carnivorous species due to their ability to metabolize amino acids derived from animal protein [34,73]. However, gut microbiota has shown great inter-individual variability and changes throughout the different developmental stages of the host that make it difficult to understand the biological function of microbes in their natural ecosystem, as the microbial physiology is not fully resolved [28,74].

The relative abundance at the family level showed a dominance of Enterobacteriaceae and Pseudomonadaceae in all the groups of our study. While it is not common to find Enterobacteriaceae as a dominant family in the digestive tract of the sole or other marine species, a high abundance has previously been reported in healthy rainbow trout (*Oncorhynchus mykiss*) and other farmed fish [75,76], which may suggest a symbiont role of this family. However, the Pseudomonadaceae was another predominant taxon in the Senegalese sole in all the experimental groups. Similarly, an important composition of bacteria of the *Pseudomonas* genus in the intestinal microbiota of Senegalese sole juveniles has been previously reported [63]. Nevertheless, *Pseudomonas* abundance can change depending on the larval stage, stocking density and the diet used [63,68,70]. 

Phylloquinone supplementation induced a decrease on the alpha diversity of the intestinal microbiota of *S. senegalensis*, in contrast to previous studies conducted in mammals. Wagatsuma et al. [77] reported an increase in Chao richness and a negative correlation with non-carboxylated osteocalcin in humans on a phylloquinone-supplemented diet. Additionally, Ellis et al. [35] found no significant difference in the Shannon diversity of the cecal microbiota of male mice when comparing the dietary inclusion of VK1 and menaquinones (MK-4 and MK9) with a VK-deficient diet. Discrepancies between present and previous studies might be related to the bacterial community studied (intestinal lumen *versus* feces) and/or the dietary content of VK included in the reference diet (non- and VK deficient diets), respectively. Furthermore, Ellis et al. [35] also showed greater Shannon diversity in female mice than in males. While sex gender might already be established in examined fish, they were not adults and were not sexually active, and therefore sexual differentiation was not considered in the present study. Nevertheless, when Senegalese sole breeders were fed with diets containing the same levels of VK1 assessed here, a different gender response was observed in the profile of plasma testosterone levels over time [19]. Therefore, an important consideration for further research on how VK1 dietary supplementation improves animal microbiome might be to evaluate the potential gender differences in adult organisms. 

In our case, phylloquinone supplementation might regulate the relative abundance of potential pathogens within the intestinal microbiota of *S. senegalensis*. Petersen and Round [78] have described dysbiosis as the imbalance or change in the microbiota when compared to a healthy individual. The loss of beneficial microbes, expansion of pathobionts, and loss of diversity are all events that can be encompassed by this term. The increased alpha diversity indices in fish fed with a VK1 non-supplemented diet might be considered under the extension of this concept. An increased abundance of potentially harmful microorganisms (*Vibrio*, *Pseudoalteromonas* and members of the Rhodobacteraceae and Campylobacteraceae families) has been revealed in specimens fed with the Control diet. The higher abundance of these microorganisms might be related to the high vulnerability to diseases observed in this fish species, probably connected with the difficulties in weaning it onto formulated diets, potentially leading to the observed high mortality rates during the weaning and post-weaning stages [2]. The main pathological problems in Senegalese sole are bacterial diseases (e.g., tenacibaculosis or photobacteriosis) and vibriosis; the last one is usually detected as secondary and primary infections [2]. *Vibrio* species can be found naturally in the microbiota of *S. senegalensis* [67,69,71], and eventually cause health damages, depending on the susceptibility of the host and the relationships between microorganisms [79,80]. While it should be considered that abundance variations of the *Vibrio* genus in fish are notably susceptible to environmental and rearing conditions [81], these results may suggest that Senegalese sole fed with a diet supplemented with phylloquinone might be less prone to suffer vibriosis outbreaks.

Rhodobacteraceae was also significantly more abundant in fish fed the Control diet than in those fed with the VK1 supplemented diet. Members of this family are commonly found in the marine environment and can participate in the degradation of organic and inorganic molecules, sulfur oxidation, anoxygenic photosynthesis, carbon monoxide oxidation and in the synthesis of secondary metabolites [82,83]. In white shrimp (*Penaeus vannamei*), its overabundance has been associated with poor health status and low body weight [84,85], while it might have the probiotic potential of reducing the influence of stress caused by low temperatures [86]. Since both dietary groups were reared at the same temperatures, its higher abundance in the non VK-supplemented dietary group might also be interpreted as a loss of health status in these specimens. 

Regarding *Pseudoalteromonas*, there is no previous record about its presence in *S. senegalensis*, however it has been found in marine hosts such as gilthead sea bream (*Sparus aurata*) and European sea bass (*Dicentrarchus labrax*) [82]. While it seems to reduce microbial competition by producing antimicrobial compounds and biofilm formation [87], it has been shown that some strains could synthesize proteases with the potential to generate virulence in marine sponges [88]. Thus, although *Pseudoalteromonas* is not an obligate pathogen, it is considered an opportunistic bacterium that has been used as an early indicator of health condition [89]. Therefore, its higher abundance in specimens fed non-supplemented VK1 diet might also be a further indication of poorer health condition than in those fed with a VK1 supplemented diet. Similarly, the Campylobacteraceae family, belonging to the Epsilon-proteobacteria class, is known to include opportunistic pathogens such as *Campylobacter* and *Arcobacter* that are generally found in organisms exposed to environments with higher fractions of opportunistic bacteria [90,91] and its overabundance has been associated with a reduction in body size, skin thickness and signs of illness in aquatic organisms [92,93]. Therefore, the tendency of fish fed VK1 supplemented diet to show a lower abundance of Campylobacteraceae might also be linked to the reduced microorganism diversity with a healthier status of the host.

Finally, *Methylobacterium*, *Roseomonas*, *Shewanella* and *Delftia* were also associated with VK1 dietary supplementation. *Methylobacterium* species are facultative methylotrophs capable of metabolizing single carbon compounds as an energy source, producing poly-beta-hydroxybutyrate and participating in the inhibition of pathogen growth [94,95]. This increased abundance of *Methylobacterium* species is in line with the reported lower abundance of *Vibrios* and Rhodobacteraceae taxa. *Roseomonas* and *Delftia* (from the Comamonadaceae family) have been reported to also be involved in the degradation of carbon compounds such as toluene and benzene [96,97]. These results might suggest that VK1 favors the promotion of these bacteria by interaction with molecules and receptors of the innate immune system [74]. Moreover, these modulations can be induced by changes in energy sources and an adaptation to survival under these specific conditions [30]. Therefore, taking the microbiome results presented here as a whole, VK1 supplementation might be a suitable nutritional approach to modulate the microbiome and reduce bacterial infections and Senegalese sole susceptibility to diseases during weaning and post-weaning stages. Nevertheless, in order to promote a better microbiome, the optimal level of VK1 supplementation in aquafeeds for Senegalese sole juveniles still remains to be determined in future research works.

## 5. Conclusions

In the present work, some insights into the biological functions of VK1 in fish species and the related mechanisms, as well as some guidelines for proper storing of aquafeeds supplemented with VK1 have been presented. The results obtained highlighted that dietary supplementation of phylloquinone contribute to balance the intestinal microbiota of *Solea senegalensis* during pre-growing, limiting the proliferation of some potential pathogens. In this sense, dietary supplementation of 1250 mg kg^−1^ of VK1 was able to increase the abundance of the Comamonadaceae family, as well as to decrease the population of *Vibrio*, *Pseudoalteromonas* and particular members of the Rhodobacteraceae family. However, for an optimal maintenance of dietary VK levels in aquafeeds, the storing temperature seemed to be critical. Feeds should be stored at −20 °C for short-term storage (7 days), while maintenance at −80 °C is recommended for the long-term (up to 3 months). Therefore, further research should be done to improve short- and long-term storage of feeds when supplemented with VK1. Since the use of refrigerators and freezers at fish farms might increase the costs of feed maintenance, increasing the amounts of antioxidants included in the feed formulation might be a suitable alternative to protect VK1 from oxidation.

## Figures and Tables

**Table 1:** Summary of sequencing output values per replicate of each dietary group and sampling time.

Sample *	Raw Sequences	Processed Sequences	%
CT0-1	113,594	23,410	20.6
CT0-2	95,040	18,880	19.9
CT0-3	103,108	22,030	21.4
CT0-4	93,905	21,211	22.6
CT30-1	190,551	36,207	19.0
CT30-2	255,262	51,304	20.1
CT30-3	87,488	17,177	19.6
CT30-4	78,162	13,946	17.8
VK30-1	100,878	20,715	20.5
VK30-2	96,232	18,334	19.1
VK30-3	69,636	14,486	20.8
VK30-4	110,180	20,667	18.8

* Numbers 1–4 represent replicates for each study group. CT0, control group at the beginning of the trial; CT30, control group fed with the diet no supplemented with VK1 at day 30 of the trial; and VK30, group fed with the diet supplemented with 1250 mg kg^−1^ of VK1 at day 30 of the trial.

## Data Availability

While sequencing data is available at NCBI SRA database (accession number PRJNA875592), data of VK1 levels in feeds are available upon request.

## Supplementary Materials

The following supporting information can be downloaded at: https://www.mdpi.com/article/10.3390/ani12233248/s1, Figure S1: Experimental design and sampling times. Figure S2: Relative abundance (%) of the overall most prevalent bacterial communities’ phyla in the digestive tract of individual *Solea senegalensis* (Proteobacteria divided at taxonomic class level).
