# Supplementary material for: Fish Microbiome Modulation and Convenient Storage of Aquafeeds When Supplemented with Vitamin K1
Source: Animals (Basel). 2022 Nov 23;12(23):3248. doi: 10.3390/ani12233248 (PMC9735498; doi:10.3390/ani12233248)
Supplement: Supplementary file 1 [file animals-12-03248-s001.zip › animals-1952269-supplementary.pdf]

Supplementary Materials:

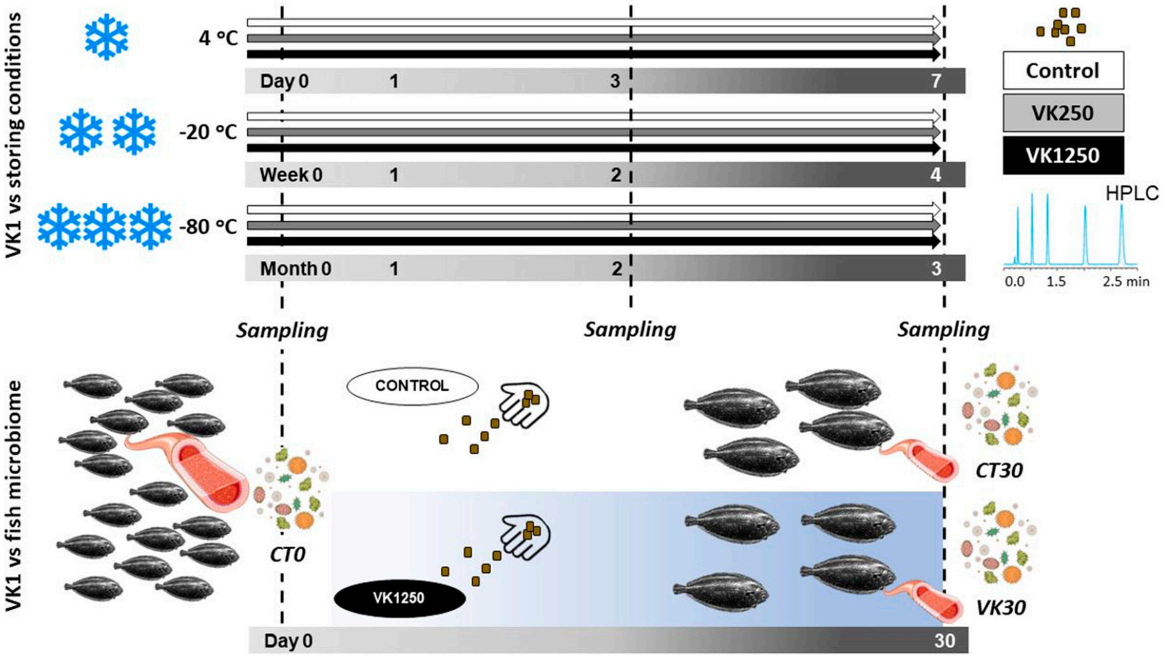

Figure S1. Experimental design and sampling times. **Top panel:** experimental design indicating sampling times for quantification of dietary VK levels under different storage conditions. Feeds were specifically formulated to contain three different levels of VK1: not supplemented with VK1, Control diet (Control); diet supplemented with 250 mg kg^{-1} of VK1 (VK250); and diet supplemented with 1250 mg kg^{-1} of VK1 (VK1250). Equal amounts of diets (5 kg) were stored at 4, −20 or −80 °C for 7 days, 4 weeks or 3 months, respectively. When stored at 4 °C, VK content in feeds was evaluated at 1, 3 and 7 days after the feed bag was opened. When stored at −20 °C, the VK content was assessed at 1, 2, 3 and 4 weeks; while at −80 °C the VK levels were evaluated at 1, 2 or 3 months. Dietary VK content was evaluated through HPLC with fluorescence detection analysis. **Bottom panel:** experimental design and sampling times for fish microbiome analysis. A total of 46 fish juveniles were randomly distributed in two flat bottom 200 L tanks (23 specimens each) connected to a single recirculating aquaculture system. Fish were fed with two experimental diets: A control diet without VK1 supplementation (CT) or a diet supplemented with 1250 mg kg^{-1} of VK1 (VK1250). The nutritional trial lasted 30 days and the fish were fed once per day with 3% daily feed intake. Four fish were sampled at the start of the experiment (CT0), while the other four fish from each experimental group (CT30 and VK30) were sampled at the end of the experiment (30 days).

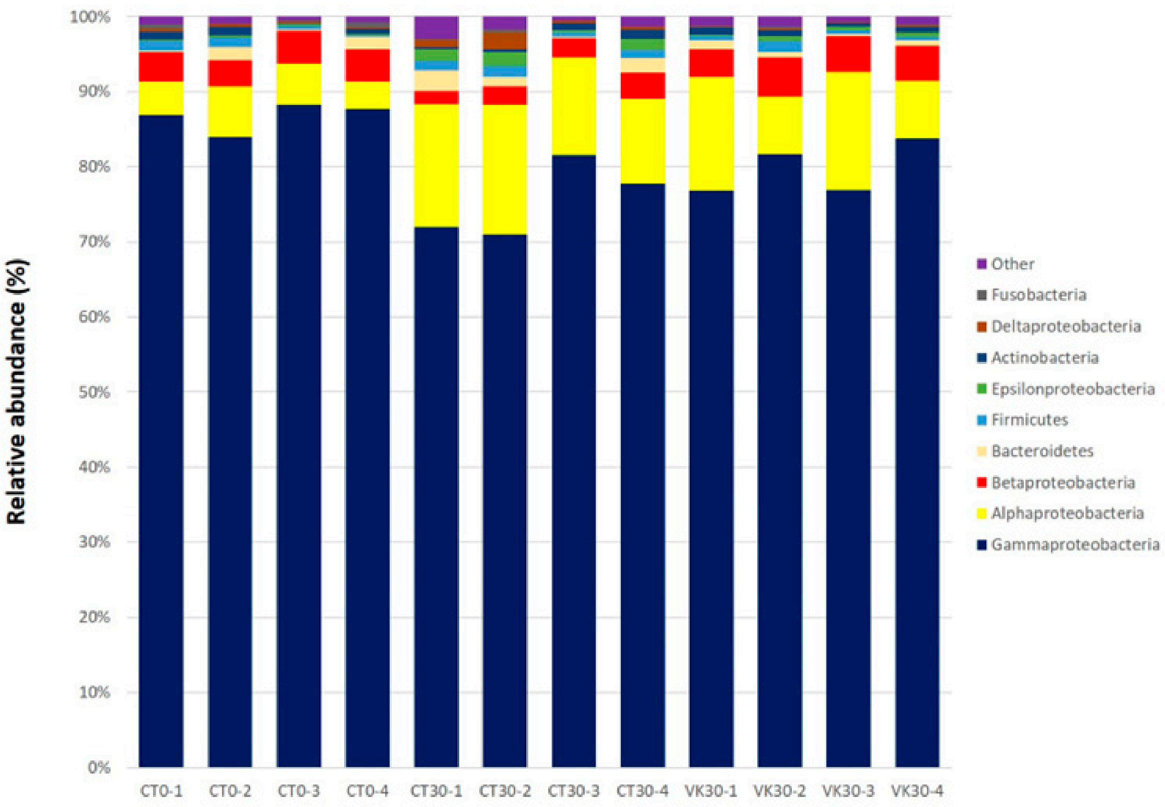

Figure S2. Relative abundance (%) of the overall most prevalent phyla in the digestive tract of individual *Solea senegalensis* (Proteobacteria divided at taxonomic class level). CT0, control group at the beginning of the trial; CT30, control group fed with the diet no supplemented with VK1 at day 30 of the trial; and VK30, group fed with the diet supplemented with 1250 mg kg^{-1} of VK1 at day 30 of the trial.
